# Assessment of Changes in the Hemoglobin Level under the Influence of Comprehensive Spa Therapy Using Therapeutic Radon-Sulfur Waters and Its Correlation with Free Radical Reactions

**DOI:** 10.1155/2020/4637129

**Published:** 2020-07-20

**Authors:** Jadwiga Kuciel-Lewandowska, Michał Kasperczak, Małgorzata Paprocka-Borowicz

**Affiliations:** Department of Physiotherapy, Medical University of Wroclaw, Wroclaw, Poland

## Abstract

**Introduction:**

Hemoglobin is a protein present in erythrocytes of higher organisms. Its main function is to transport oxygen from the lungs to tissues and carbon dioxide from tissues to the lungs. Hemoglobin contains Fe^2+^, catalyzes free radical reactions, and may initiate oxidation reactions by enzymatic and nonenzymatic degradation. The aim of the study was to evaluate the effect of balneophysiotherapy on the hemoglobin level in osteoarthritis patients and to try to assess the association of those metabolic changes with free radical reactions.

**Materials and Methods:**

The study was conducted in Przerzeczyn-Zdrój spa resort. It included patients receiving spa treatment over 21-day sessions. The studied group consisted of *n* = 122 patients with joint and back pain due to osteoarthritis or disc herniation. Their age ranged between 32 and 67 years with a mean age of 53.5. Blood samples were collected before treatment and after 21 days at the spa. Standard tests were used. The results were statistically analyzed using the sign test and the Wilcoxon test.

**Results:**

In the study group, we observed a drop in the hemoglobin level following spa treatment. Before treatment, the mean hemoglobin level was 14.1549 g%, and after treatment, it was 14.0008 g%.

**Conclusions:**

(1) In the study, we concluded that balneophysiotherapy in osteoarthritis patients resulted in a decrease in the mean hemoglobin level. (2) The decrease in the mean hemoglobin level in osteoarthritis patients treated at the spa resort may indicate an association with free radical reactions. This trial was registered with NCT03405350.

## 1. Introduction

Hemoglobin is a protein present in erythrocytes of higher organisms. Its major function is to transport oxygen from the lungs to tissues and carbon dioxide from tissues to the lungs. It is composed of four polypeptide chains forming similar pairs (two alpha chains and two beta chains) as well as four heme prosthetic groups. Heme contains Fe^2+^ ions, which, in the presence of oxygen, are oxidized to Fe^3+^. In addition to the formation of a heme-Fe^3+^ complex, a superoxide radical O_2_^−^ is produced [1]. On the other hand, in the presence of other electron donors such as nitrates and aminophenols, a double-electron reduction of hemoglobin-bound O_2_ can occur with the formation of H_2_O_2_. Both superoxide and hydrogen peroxide are classified as reactive oxygen species (ROS). Both compounds are by-products of many aerobic reactions. As a result of further reactions, the hydroxyl radical OH^−^ is created, which is even more reactive. Initially, Fe^2+^ is oxidized in the presence of H_2_O_2_ (Fenton's reaction) followed by regeneration of the Fe^2+^ ion [2]. By combining both reactions, the so-called Haber's reaction is formed, which is catalyzed by Fe^2+^/Fe^3+^ ions:

(1) $$\begin{array}{c} {\text{O}}_{2^{-}}+{\text{H}}_{2}{\text{O}}_{2}⟶\text{OH}+{\text{OH}}^{-}+{\text{O}}_{2}. \end{array}$$

The iron ions (Fe^2+^/Fe^3+^) attached to proteins such as hemoglobin can catalyze the reactions shown above [1]. There are cell-protective mechanisms against ROS. They consist of enzymes catalyzing O_2_^−^ and H_2_O_2_ breakdown. In healthy humans, around 3% of hemoglobin can be oxidized to methemoglobin daily, which, in turn, cannot bind to oxygen. This reaction is the main source of superoxide [2,3]. Methemoglobin reductase catalyzes reduction of methemoglobin to hemoglobin and indirectly inhibits ROS formation. It also has been established that heme-containing proteins can, under certain circumstances, inhibit ROS synthesis, hydroxyl radical, in particular, in the presence of low-molecular-weight antioxidants, e.g., ascorbic acid [4]. Due to their structure and function, erythrocytes are sensitive to reactive oxygen species [5]. As can be observed, hemoglobin with Fe^2+^ contained in erythrocytes can catalyze free radical reactions, and as a result of enzymatic and nonenzymatic heme decomposition and loss of heme iron, the products can also initiate oxidation, which is essential for ROS generation. Hemoglobin is not only involved in auto-oxidation but can also be exposed to superoxides resulting from activation of other blood cells and oxidation of catecholamines and xenobiotics [6, 7]. In the second stage, superoxide dysmutation leads to the formation of hydrogen peroxide, which takes part in heme degradation, followed by synthesis of soluble biliverdin further creating nonsoluble bilirubin and carbon monoxide in the presence of iron ions [8].

The oxygen free radicals, also called the reactive oxygen species (ROS), are atoms, molecules, or moieties with independent activity, which also contain one or more unpaired electrons in the valence shell. Those electrons determine the reactivity of the free radicals and initiate redox chain reactions, which is not favourable. The main source of free radicals in the body is the cellular respiration catalyzed by various enzymes. The free radicals are balanced by antioxidants, which are present in low concentration but significantly inhibit oxidation [9]. ROS includes singleton oxygen ^1^O_2_, superoxide O^−^_2_, hydrogen peroxide H_2_O_2_, and hydroxyl radical OH [10]. In health, the free radical level is strictly controlled keeping balance between formation and degradation of reactive oxygen species. The imbalance leads to oxidative stress. The oxidative stress is when the antioxidant drops or when the ROS production is increased for various reasons [11]. As long as there is a balance between ROS formation and elimination, they are harmless for the body. The imbalance leads to manifestation of the toxic effect of ROS, including inflammatory disorders of the muskuloskeletal system. It is followed by a cascade of enzymatic reactions leading to hialuronic acid depolymerization, loss of tissue elasticity, proteoglycan and collagen degradation, protein oxidation and inhibition of chondrocyte proliferation [12]. Free radicals also take part in pathogenesis of other disorders such as atherosclerosis, neurogenerative diseases including Alzheimer's or Parkinson's disease, inflammation, allergy, cancer, diabetes, and macular degeneration [13, 14]. The human body has a couple of mechanisms regulating (limiting) production of free radicals. The antioxidation system consists of antioxidants. The antioxidation system includes:

(1) Endogenous antioxidants produced by the body:
  - Enzymatic: superoxide dysmutase (SOD), glutathione peroxidase (GSH-Px), and catalase
  - Nonenzymatic: linolenic acid, polyamides, albumin, bilirubin, glutathione, uric acid, ceruloplasmin, transferrin, and coenzyme Q10, with different targets
(2) Exogenous antioxidants consumed with food: vitamins C, A, and E, carotenoids, xantophiles, and polyphenols. They indirectly take part in the free radical reaction affecting cellular signalling pathways, activity of enzymes and genes engaged in apoptosis, and DNA repair [15].

The aim of the study was to evaluate the effect of balneophysiotherapy on the hemoglobin level in osteoarthritis patients and to try to determine whether this effect is associated with free radical reactions.

## 2. Materials and Methods

The study was conducted at the spa resort in Przerzeczyn-Zdroj on patients receiving spa treatment during 21-day sessions. The patients' blood samples were collected from the ulnar vein using a vacuum tube before treatment and after 18 days. The blood samples were centrifuged, and the plasma was kept at +6°C before testing. The patient group consisted of *n* = 122 patients with peripheral and spinal osteoarthritis, including 91 females and 31 males aged 32 to 67, with the mean age of 53.5. The main inclusion criterion was the presence of peripheral and/or spinal osteoarthritis and no contraindications for complex spa treatment. The patients above 80 years were excluded. Most patients received normal or light diet, mainly containing cooked dishes with low fat content. Both diets had normal caloric intake. The patients were offered a series of 10 sessions of each type of intervention depending on their needs and diseases. The treatment options included sulfide-radon baths, peloid compresses, therapeutic exercises in groups and individually, biostimulation with lasers, and interferential current. The complex spa treatment included the following:

- Sulfide-radon baths including whole body or only upper and/or lower extremities at the temperature of 37-38°C for 20 min
- Partial peloid compresses, time 20 min, and temp. 40–42°C
- Therapeutic exercises in the swimming pool with neutral water
- Individual exercises with equipment or group exercises selected for each patient depending on his or her fitness; mean duration 30–45 min
- Field therapy: walking and outdoor exercises
- Dry massage of the cervical (CC), thoracic (TH), or lumbosacral (LS) spine;
- Laser therapy: parameters: pulse or continuous mode, wavelength 808 nm, power 12.0 J, 400 mV, and duration 30 s
- Low-frequency magnetic field: duration 20 min, square impulse, induction 5 mT, and frequency 20–50 Hz
- Ultrasound therapy: parameters: probe 800 kHz/6 cm^2^, impulse waves: 2 ms impulse, 9 ms break, at the dose of 0.5–0.6 W/cm^2^ over 6 min
- Cryotherapy: ventilation, duration 2–3 min and temp. between −80°C and −110°C
- Electrotherapy: Berndard's didynamic current, parameters: DF1 CP4 LP4, Nemec's interferential current (frequency 0–100 Hz), transcutaneous electrical nerve stimulation (TENS): square wave, impulse 0.2 ms, frequency 40 Hz, and regulated current 0–100 mA
- Phototherapy: Sollux lamp with a blue filter, distance 30–40 cm; duration 15 min and Bioptron lamp–distance 10 cm; duration 5–10 min

Particularly important was, unique in Europe, the healing water at Przerzeczyn-Zdroj spa.  Table 1 shows the physical and chemical properties of the water.

The chemical composition characteristic for Przerzeczyna-Zdroj water was within the ranges established over years of observation.

The study design included a control group, which consisted of 14 individuals selected from the resort staff, including 10 females and 4 males aged 24 to 58 with the mean age of 41.7. Healthy nonsmokers and nondrinkers were included in the control group. They were advised to carry on their usual lifestyle and were asked not to use any of the treatment options available at the resort.

The study was approved by the Bioethics Committee of Wroclaw Medical University, Resolution No. KB-401/2008, as well as by the Head of Uzdrowisko Przerzeczyn Sp. z o.o., and each patient gave his or her informed consent. The documents are in the author's possession.

The results were statistically analyzed using the STATISTICA 9.1 and Microsoft Excel 2007 software. For statistically significant differences, the detailed information was obtained by descriptive statistics, comparing medians, means, and quartiles. The differences before and after treatment were evaluated in each group using the Wilcoxon test and the sign test. The significance level was set at *α* = 0.05.

## 3. Results

**Figure 1:**
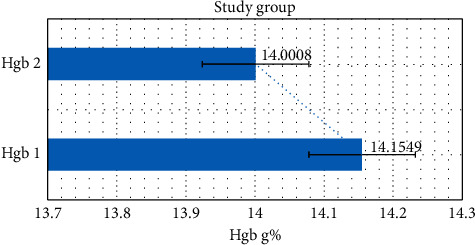
The change in the mean Hgb level before and after treatment in the studied group.

In the studied group, we observed a drop in the hemoglobin level. The mean hemoglobin level before treatment (HGB1) was 14.1549 g% and 14.0008 g% after treatment (HGB2) Figure 1.

The decrease in the hemoglobin level was statistically significant *p*=0.005 in both tests.

The descriptive statistics are shown in Table 2.

**Figure 2:**
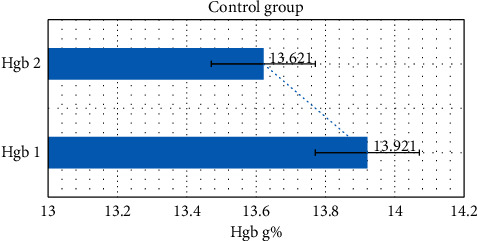
The change in the mean Hgb value before and after the observation period in the control group.

In the control group, the mean Hfb level decreased from 13.9210 g% before treatment (HGB1) to 13.6210 g% after treatment (HGB2).  Figure 2.

For the control group, we used the same tests to compare the results. In Wilcoxon test, *p*=0.058, while in the sign test, *p*=0.092, which means that the differences are not statistically significant. The median and mean also indicate a negligible drop in the Hgb level after the observation in the control group. The descriptive statistics for the control group are shown in Table 3.

## 4. Discussion

In our study, we observed a statistically significant drop in the hemoglobin level after treatment in patients receiving spa treatment. We searched scientific databases and found studies evaluating the effect of balneotherapy on the hemoglobin level. Different outcomes have been reported, usually an increase in the hemoglobin level. Durda et al., in their study on water rich in sulfur and hydrogen sulfide, showed an increase in the hemoglobin level. The authors believe that the baths stimulate redox reactions, which is indicated by an increase in the hemoglobin level and erythrocyte count [16]. Misztela et al. also observed a rise in the hemoglobin level in patients with rheumatoid arthritis receiving artificial sulfur and hydrogen sulfide baths [17]. Frih et al., in their study on dialysis patients receiving kinesiotherapy, observed no change in the hemoglobin level [18]. The study by L Xu et al. showed that balneotherapy resulted in an increase in the erythrocyte count, hemoglobin level, and hematocrit [19]. Jokić et al. showed that treatment with sulfur and peloid baths in patients with hip and knee osteoarthritis led to a significant reduction in plasma lipid peroxidation and changed plasma activity of superoxide dysmutase and catalase. A significant increase in the hemoglobin level was observed, which was assumed to be a result of inhibited production of excess free radicals [20]. The study by Zinchuk et al. showed that dry sauna causes a drop in the hemoglobin level. A single visit to the sauna causes oxidative stress, and its symptoms are less pronounced after repeated exposure to heat [21]. In addition to a decrease in the hemoglobin level, our study showed an increase in the total antioxidative level and changes in specific components of the endogeneous antioxidation system, i.e., decease in the albumin and bilirubin level, as well as an increase in uric acid concentration, suggestive of free radical reactions [22, 23].

The activation of free radical reactions is the body's response to the balneotherapeutic stimulus. Later, the accumulation of balneotherapeutic stimuli causes metabolic shift and activation of defense mechanisms. The antioxidant production increases. The end result is an increased total antioxidant level, activation of adaptive processes, and regeneration dependent on largely unexplained functional and structural changes associated with the autonomous system activation. This mechanism can be a foundation for positive systemic changes leading to improved health after spa treatment [24].

There have been various and contradictory outcomes reported, depending on specific comorbidities and types of intervention. As far as healing water is concerned, the metabolic response depends on the chemical content and pharmacodynamic properties of the water. A significant effect (increase in hemoglobin) was observed in response to sulfur-rich water having particular metabolic activity. So, how to explain the decrease in the hemoglobin level in patients at Przerzeczyna-Zdroj? Generally speaking, a lower hemoglobin level is not a desirable outcome. It can be presumed that radon is a strong stimulus (stressor), activating a free radical reaction in the body. The main activating element responsible for metabolic changes is thought to be low-dose alpha radiation. The main mechanism might be radiation hormesis activating hemoglobin. Hemoglobin is mainly responsible for oxygen transport, but it also catalyzes free radical reactions and inhibits ROS formation [25].

Contradictory results by other authors indicate that further multidisciplinary studies evaluating the full blood count, heme degradation products, and iron metabolism are necessary. Because we were unable to clearly determine factors responsible for different results, randomized clinical trials on large groups of patients receiving isolated forms of treatment are necessary. Many factors affect the hemoglobin level. The changes in the hemoglobin level cannot always be easily interpreted. Evaluation of hemoglobin solely as a part of free radical reaction is not enough for the assessment of the antioxidation system; however, both are closely related. The observed changes signal metabolic shifts in response to balneotherapeutic stimuli.

## 5. Conclusions

1. In this study, we concluded that balneophysiotherapy in osteoarthritis patients resulted in a decrease in the mean hemoglobin level
2. The decrease in the mean hemoglobin level in osteoarthritis patients treated at the spa resort may indicate a correlation with free radical reactions

## Figures and Tables

**Table 1:** The results of physical and chemical testing on 24.04.2008 of the healing water at the spring and the natural therapy resort (NTR), our own source.

No	Sample location	Water temp. in (°C)	pH	The content of the ingredient in 1 dm^3^ of water			
				H_2_S Mg	HCO_3_ Mg	Rn NCi	Rn Bq
1.	Hole no II	12.0	7.62	1.96	263.2	2.21	81.8
2.	Hole no IX	12.0	7.72	1.70	289.6	1.71	63.3
3.	Tub MSP	16.0	7.65	1.87	277.9	2.20	81.4

**Table 2:** Descriptive statistics: study group.

HGB	*N*	Mean	Standard deviation	Minimum	Maximum	Percentiles		
						25.	50. (median)	75.
HGB1	122	14,1549	1,2926	9,9000	17,4000	13,2000	14,1000	15,0000
HGB2	122	14,0008	1,3095	9,8000	17,4000	13,1000	14,0000	14,8000

**Table 3:** Descriptive statistics: control group.

HGB	*N*	Mean	Standard deviation	Minimum	Maximum	Percentiles		
						25.	50. (median)	75.
HGB1	14	13,921	1,2071	12,1000	15,9000	13,1500	13,4000	15,0500
HGB2	14	13,621	0,9267	12,0000	15,1000	12,9700	13,5000	14,5000

## Data Availability

All data are contained and described within the manuscript. The datasets used and/or analyzed during the current study available from the corresponding author on reasonable request. The number of clinical trial is NCT03405350. All information about this study is available under this number.
